# Unveiling the master narratives of a sample of STEM students at an urban public college in New York City

**DOI:** 10.3389/fsoc.2025.1661399

**Published:** 2025-10-10

**Authors:** Krystal Reynoso, Fadel Ismail, Daniela Ávila, Maria Isabel Roldós

**Affiliations:** ^1^Lehman College, Bronx, NY, United States; ^2^CUNY Institute for Health Equity, Bronx, NY, United States

**Keywords:** STEM, MSI, immigrant students, narratives, NYC 4

## Abstract

This brief research report presents how rigid STEM narratives are perceived by undergraduate students from diverse backgrounds at an urban Minority Serving Institution (MSI) in NYC. This research focuses on student voices and reveals the unique challenges, motivations, and strengths they bring to STEM. A total of 15 peer to peer qualitative interviews were conducted between May and December of 2024. Content analyses using an inductive approach was used to cross code between the interviewers and student participants using content analyses revealed key themes such as: the importance of time management support, diverse mentorship, the role of parents and family members, among others. Findings illustrate that representation and authentic support significantly impact student success. Recommendations to address barriers and proposals of supportive solutions designed to break down STEM stereotypes to serve undergraduate students from diverse backgrounds.

## 1 Introduction

Scientific advances are fueled by diverse perspectives. U.S. philosopher Thomas S. Kuhn proposed in his iconic book, *The Structure of Scientific Revolutions* (1962) (Kuhn, 2012), that science advances are stifled when paradigms that guide the scientific query are unchallenged (Ferrini-Mundy, 2013). In fact, he argues: “science advances results from the ‘development-by-accumulation' of accepted facts and theories” (Kuhn, 2012; Kindi and Arabatzis, 2013). To this end, diversity in perspectives has the potential to increase the scientific quality of arguments and spark a stronger thought toward scientific innovation.

The voices of diversity in 2025 are very much different than those in 1965 when Kuhn proposed this hypothesis. The U.S. Census Bureau estimate the nation's population is close to 345 million people in 2025, which is a 2.93% increase since Census Day on April 1, 2020 (US Census Bureau, 2024). Migration continues to be a key driver of population growth, in addition to a higher birth rate among migrants (Camarota and Zeigler, 2021). Highly skilled immigrants (first- or second-generation) in STEM fields may provide a competitive edge and may increase the innovative capacity of the United States. In 2019, immigrants comprised 28% of the college-educated STEM workforce, with over 45% holding STEM doctoral degrees (National Center for Science and Engineering Statistics, 2023). The value of diversification of academics in STEM is crucial and warranted from different yet interlinked perspectives such as social justice, economic competitiveness, and scientific quality (Xie, 2024).

Minority serving institutions (MSIs) play a crucial role among first- and second-generation immigrant background students to earn a baccalaureate STEM degree, but not necessarily to earn a doctoral degree in STEM. A review of the InfoBrief from National Center for Science and Engineering Statistics in 2023 indicate that Black, Hispanic, and AIAN doctorate recipients from 2010 to 2020 disproportionately earned a bachelor's degree from minority-serving institutions [National Science Foundation (NSF), 2023]. Therefore, it is crucial to learn what MSIs can do to promote post-baccalaureate education among first- and second-generation immigrant background students.

This study is a qualitative investigation of the perceptions, motivations, and barriers to enrollment and graduation among a sample of currently enrolled students in STEM-related majors in a MSI urban campus in New York City.

## 2 Methods

This project recruited 15 currently enrolled STEM students from Biology, Chemistry, Mathematics, Computer Science, and Health Sciences to explore perceptions, motivations, and barriers to enrolling, retention and graduating in STEM-related programs. This project received IRB approval number 2024-0544 and Title: Understanding perceptions, motivations and barriers to enrolling, staying enrolled and graduating in STEM careers among Lehman college students.

### 2.1 Research framework

The praxis of inquiry is Intersectionality. Intersectionality is a sociological framework that analyzes how various systems of power and oppression, such as racism, sexism, and classicism, overlap and intersect creating unique and multifaceted experiences of social inequality (Taj and Ashraf, 2024). It emphasizes that an individual's identities, like race, gender, and socioeconomic status, collectively shape their lived experiences (Carter and Seaton, 2025). Furthermore, intersectionality unveils how to operatively manage cultural complexities (Hays, 2024) and understand forms of discrimination that may result in disadvantages for those with overlapping marginalized identities (Taj and Ashraf, 2024; Collins and Bilge, 2016). Using intersectionality as the framework for inquiry, this study conducted qualitative semi-structured interviews to explore student's perceptions and considerations of enrolling in STEM careers, as well as barriers and incentives to remaining enrolled and graduating in these fields.

### 2.2 Recruitment

The recruitment was done using peer referrals. The principal investigator of this project recruited 3 STEM students as research assistants (RAs) for the NSF grant titled “Collaborative Research HSI_HUBs: Intersectionality in STEMs.” Each RA recruited between 4 and 5 peers in their field to interview. The inclusion criteria for the study were that students were required to be currently enrolled in a STEM-related field at the MSI campus and being over the age of 18 years. Consent to participate was collected at time of recruitment.

The recruitment used a purposive, peer-referral sampling led by three undergraduate RAs who each recruited peers in their field. Interviews were conducted on Zoom between Summer and Fall 2024, in English, averaged ~60 min, and participants received a $20 Amazon e-gift card.

#### 2.2.1 Sample size and saturation

The research team judged the sample size as adequate when thematic saturation was reached. Interviews were conducted mainly during the summer months with less availability of students on campus. To this end, 4-5 interview per interviewer in the STEM discipline proved to be sufficient to capture student's narratives. The research team cross-referenced the reading of the narratives by reading each other's interviews. The research team discussed the emerging themes. If themes were not identified, additional interviews were conducted (one per RA) to confirm that no new themes appeared. On this basis, 15 interviews were sufficient for the goals of this project.

### 2.3 Semi-structured Interviews

The semi-structured interviews followed a research protocol that mimics a conversation between two-peers to seek to collect a “narrative” of storytelling that describe students' experiences. Interviews were conducted via zoom during a time agreed between the RA and the student. No individual identifier information was collected. Participants used a pseudo-name during the interview and cameras-on were optional. The intersectional variables associated with socioeconomic status were gender, age, family income, race or ethnicity, family support, place of birth, nativity, and school or degree program at the College. RAs took brief field notes after each session to capture contextual details and early analytic memos.

Based on findings of the literature on intersectionality, higher education and from extensive discussion between the RAs of the study, the following narratives and counter-narratives in STEM education were explored in the semi-structure interviews:

- STEM is for White and Asian Men (Allen et al., 2022).
- STEM comes from Innate Geniuses (Covarrubias et al., 2019).
- STEM is a Pure Meritocracy (Weston et al., 2019).
- STEM is a “Weed-Out” Culture (Weston et al., 2019).
- STEM is Objective and Apolitical (Allen et al., 2022; Rodriguez et al., 2017).
- STEM is Separate from Culture and Identity (Rodriguez et al., 2017).

The methodology entailed discussing the STEM stereotypes described above through the lens of the participant's lived experiences. To this end, participants explored the following:

- How do students at an urban MSI perceive STEM stereotypes?
- What challenges and opportunities do they encounter in STEM fields?
- How does the MSI environment influence their STEM identity and persistence?

### 2.4 Data analysis

The data analysis used inductive thematic analysis. Best practices from step-by step Qualitative content analysis was used (Mayring, 2021). The three undergraduate RAs generated data-driven codes in Excel/Word, then met to build a shared codebook, compare interpretations, and refine theme boundaries. All transcripts were consensus-coded; disagreements were resolved through discussion. We enhanced trustworthiness through regular analytic meetings and peer debriefing with the faculty mentor, and by keeping brief notes on coding decisions. Intersectionality provided the interpretive lens for relating emergent themes to gender, race/ethnicity, immigrant background, and socioeconomic status.

## 3 Results

A total of 15 semi-structured qualitative interviews were conducted, and close to 50% of all interviews were from students in Biology and Chemistry majors. Interviews were centered around their perceptions, and barriers to enrolling, staying enrolled, and graduating. Results revealed significant insights regarding the need for an intersectional approach to identify the challenges among STEM students and provide a framework to propose solutions to address these challenges.

Specifically, this project focused in identifying factors that encircle marginalized minority groups and their experience in STEM. The themes identified in the review of the interviews include:

3.1 Ancestry and Background (Motivation and Family Sacrifice).
3.2 Family and Social Environment on Career Choices.
3.3 Challenges Time Management and Balancing Responsibilities.
3.4 Expectations of STEM students.
3.5 Exposure/Accessibility to STEM-related Extracurriculars; Mentorship and Outreach; Academic support.

To illustrate the importance of each themes identified, selected quotes are presented in the sections that follow.

### 3.1 Ancestry & background (motivation and family sacrifice)

Under this category, two sub-themes were identified: (1) *Parental Sacrifices and High Expectations* and (2) *First-Generation College Experience and Lack of Parental Guidance*. Each of the following quotes exemplifies students' sentiments and the role of their ancestry in choosing a career in STEM.

Participant 103: “*Essentially, as a first-generation college student, parents don't really know what college means, and the value of college. As all they can really see is, as long as you have a job, that's all that really matters to us.”*

Participant 201 - “*They [my parents] have very high expectations for me because, you know, they sacrificed everything to come here so that their children could have the life that they didn't have, so they always make sure that I'm doing well in school, and they… made it clear to me that education is number one.”*

This finding is well within the literature of the expectancy-value model of motivation achievement which posits that motivation is influenced by social-cognitive variables such as self-efficacy and beliefs about the usefulness or utility of task (Dotterer, 2022). In this case, the self-efficacy of students is proven in the quotes of the participants of this study. Students expressed the usefulness of their sacrifice as perceived by their parental figures as the most useful. Research supports that parent involvement can promote children's self-efficacy which is important for having STEM interest and persisting in a STEM field (Amarnani et al., 2018). This model applied in this study helped to understand the factors that contribute to racial/ethnic minority adolescents' STEM interest and achievement, suggesting that parental involvement was the most predictive of the variables, as linked to self-efficacy. Therefore, parental involvement in STEM helps adolescents to feel more confident in their STEM abilities but it does not necessarily contribute to adolescents' STEM utility values (Dotterer, 2022; Amarnani et al., 2018).

### 3.2 Family and social environment on career choices

The semi-structured interviews revealed six sub-themes related to family and culture. Specifically, participants described differences in expectations based on both culture and gender. Students emphasized the importance of family or community members as role models, particularly those with exposure to STEM careers or knowledge, as key motivators for entering or staying in STEM fields. Peers, faculty, or mentors were mentioned less often in influencing college or major choices. A few participants, however, highlighted their peers as role models. Table 1 illustrates each sub-theme with a participant's quote.

**Table 1:** Family and social environment on career choices themes and participant's quotes.

**Sub-theme category**	**Participant's quote**
Family stressors	“*So, I think I put too much on my plate, and I try and do all these different things at once, and so it just gets stressful. Then I'd have my mind in too many different places. Not only am I a double major, but I also work about 20 h a week, and then I work in tutoring on campus. So, students see me in the library when I'm trying to study, and people come up to me. You know this too, but it's just hard to say no. I like to help people when I can, and I suck at saying no, so I like to give all my time away, and then it leaves less time for me to study. So, not having enough time for myself really demotivates me. It puts me in a depressed mindset. “*
Cultural and gender expectations	“*As far as they tell me, they‘re very proud of me, but they're always telling me not to do it because, as a woman, it's going to be very difficult for me to do that and take care of the house.”*
Role models and family influence on stem motivation	“*My uncle, actually, he's an engineer. And that kind of inspired me, because I think he's the only one that went to college on my mom's side of the family. So that really inspired me to pursue a degree in STEM.”*
Exposure to STEM through family members	“*The people I surround myself with. A lot of them are STEM-oriented... we have similar interests, and when other like-minded individuals are working toward it, it makes you want to work toward it as well.”*
Peer influence and support networks	
Role of professors, advisors, and mentors	“*Some professors go out of their way to make sure you understand the material. My organic chemistry professor, in particular, was a huge help. I went to every office hour.”*

A systematic narrative review of 55 articles that assessed the effects of role models on students' STEM motivation as a function of several features of the role models (their perceived competence, their perceived similarity to students, and the perceived attainability of their success) and the students (their gender, race/ethnicity, age, and identification with STEM) found that students and models need to be carefully studied (Gladstone and Cimpian, 2021). There is no one-size fits all. The majority-group of STEM role models are less-than-effective motivators for marginalized students because they highlight the stereotypes differences and may conclude in students feeling unwelcomed. In contrast, underrepresented-group STEM role models may not activate similar threats in majority-group students, who are generally unlikely to question their place in STEM and might therefore see the role model simply as an inspiring adult whose success they can try to emulate (Gladstone and Cimpian, 2021). Therefore, what role model is more applicable to the students from this study is differentiated by gender than race, with a note that boys or young men from a minority or immigrant background are knowingly different. In that sense, role models from underrepresented groups that apply more than one motivator may result in a net motivation gain on average and avoiding sticking to a single motivation model (Gladstone and Cimpian, 2021; Tal et al., 2024).

### 3.3 Challenges in time management and balancing responsibilities

Most of the students expressed challenges related to time management and work-school-family balance. Three sub-themes were identified: overall sentiments of an overwhelming life, job-related burnout and procrastination, and difficulties managing the time demands of STEM coursework (Table 2).

**Table 2:** Challenges time management and balancing responsibilities and participant's quotes.

**Sub-theme category**	**Participant's quote**
Sentiments of an overwhelming life	“*I was taking 18 credits, and I was working 2 jobs, and it was crazy. I was working at Staples, and then I was also tutoring, and it was just a lot... I would have class one day, and then I would leave and do my shift at Staples, and then... tutoring right after.”*
Jobs, burnout and procrastination	“*Both of [my jobs] take time, and one of them takes more brain energy than the other... but still, sometimes it takes a toll on your mind.” “Sometimes midterm season, final season with all the tutoring and stuff, I felt like my brain was fried... you're processing so much information, teaching information, but then you gotta study for another exam.”*
Managing STEM coursework takes significant time	“*Understanding the course itself is quite difficult. Chemistry is not exactly easy... if you're a student who works and does a lot of other things, it's hard to keep balance.”*

These sentiments are not unique to students in this study. Student stress and anxiety are frequently cited as having negative effects on students' academic performance (Hsu and Goldsmith, 2021). In fact, stress and anxiety are known as major challenges facing college students with more than one-third of all college students report experiencing stress and impact their academic performance within the past academic year (Morey and Taylor, 2019). Some of the research suggest that instructors and professors can play a role in mitigating these challenges (Hsu and Goldsmith, 2021). Evidence-based strategies that instructors can employ span a range of approaches, from modifying instructional techniques to empowering students with different mindsets and tools that they can use to alleviate stress (Morey and Taylor, 2019; Myers et al., 2012).

### 3.4 Expectations of STEM Students by STEM students

Participants in this sample expressed that they experience high, often unrealistic expectations as STEM students. These include overworking through extracurricular activities and feeling pressured to apply to competitive programs or internships. Some of the quotes include:

“*Doing extracurriculars, in addition to my college program, I get to meet a lot of people. I get to network... I still feel like I need to do more volunteering, more research to get into med school—that's my goal.”*

“*I expect a lot from myself because I know what I'm capable of... Even if I struggle, I feel like I have to keep proving that I belong here.“*

“*I think I can definitely like, create a lot of pressure for myself... I also doubt the ability to get where I want to go... those doubts, and also those comparisons can oftentimes... shoot me down.”*

This finding is aligned with the literature of college students in STEM disciplines in that they are increasingly faced with highly competitive and demanding degree programs and are at risk of academic overconfidence (Hall and Sverdlik, 2016). Due to this risk, students report that having high ability in science disciplines are also likely to experience academic pressure and associated physical and psychological distress (Webb et al., 2002). Important to this research is to identify the factors that may contribute to racial disparities in STEM participation beyond academic preparation related to added stress or unrealistic expectations. Some research suggest that first-year STEM students tend to overestimate their performance in general, and subsequent analyses indicate that students who overestimate are more likely to switch out of STEM (Park et al., 2023) and some race-based differences in overestimation can be explained by pre-college academic and high school a student attended (Park et al., 2023).

### 3.5 Exposure/accessibility to STEM-related extracurriculars; mentorship and outreach; academic/communal support

Themes related to the university infrastructure, including faculty mentorship and research capacity, in addition to peer- and community-environment were identified as important factors to remaining in a STEM major. Table 3 illustrates each of the sub-themes in this category.

**Table 3:** Exposure to STEM extracurriculars and mentorships and participant's quotes.

**Sub-theme category**	**Participant's quote**
Strong mentorship and academic support exist, but are underutilized	“*I think for the most part, the professors are really good. They care a lot about the students and how they perform in their class. You can tell it's not just because they want a good rating or review, it's because they genuinely want you to succeed.”*
Research and extracurricular opportunities are available but need more awareness	“*More offers for research. I think a lot of students like research, more so than maybe the actual classes and stuff... I think all departments should offer more opportunities for research and really advocate for them.”*
Some students feel a strong sense of community	“*I'm very comfortable here. It's close to my work and house, but it's not only that. I'm comfortable with the professors that I've had. I'm comfortable with my peers. I've created a good group here.” “… We're all struggling with the same things, either with coursework, life, or just work in general. So, I feel like we can all get along and bond on that aspect.”*
Students rely on peer networks for academic success	“*I like to surround myself with peers that have more effective studying techniques... The way I study best is around people. If I study alone, I feel like I'll only study what I know. I like to take from what other people do.”*

Mentoring initiatives for undergraduate and graduate students is vast, and there are many programs that support students from a minority background (Nkrumah and Scott, 2022). Under the intersectionality framework mentoring programs must include multiple intersections. This manifold approach call for the attention that understands power, challenges social context, and interrogates oppressive structures and mentors and mentees, as well as other individuals in higher education (e.g., staff and administrators) addressing hierarchies (Nkrumah and Scott, 2022).

## 4 Discussion

Family members are the most important factors to enroll in a STEM major, while their peers and supportive institutional networks were identified as the most important to remain in a STEM major. Most students expressed challenges related to time management and work-school-family balance as the main challenge to staying in a STEM career. The narratives of high expectations for students in STEM majors were upheld by those in the sample to the point where participants expressed a risk for burnout.

Specific recommendations to each of the issues identified in the themes above are detailed below into 5 categories. These categories were adapted from Hsu and Goldsmith (2021):

1. Learning and preparing to act.
  – **Leverage Family Influence for STEM Motivation**. Schools can strengthen mentorship initiatives by connecting students with professionals from similar backgrounds to reinforce STEM career possibilities.
  – **Promote peer mentoring programs**. Many students benefit from study groups and peer mentoring, but not all students know how to access these resources. Schools should promote structured peer mentoring programs for students in challenging STEM courses.
2. Connecting with students.
  – **Provide Gender-Specific Support in STEM**. Programs aimed at empowering women in STEM should address traditional barriers and provide mentorship opportunities.
  – **Need for More Flexible Scheduling and Support**. Colleges should offer more flexible class options (online, evening classes) to help students balance work and school. Furthermore, study groups and peer-led tutoring should be encouraged to help students manage heavy STEM coursework.
3. Building an empowering atmosphere in the classroom.
  – **Provide First-Generation Support Services**. Colleges should provide tailored support to first-generation students, including guidance on navigating academic expectations and career paths.
  – **Provide Financial Aid Awareness and Assistance**. More outreach is needed to help students and families understand financial aid options and scholarships.
4. Reducing testing anxiety.
  – **Provide Culturally Inclusive Career Counseling**. Advising programs should address family expectations and career misconceptions, especially for those in fields requiring extended education.
  – **Expand Time Management and Mental Health Support**. Workshops on study skills, time management, and stress reduction should be promoted. Colleges should provide more awareness about mental health resources for burnout prevention. Students also deal with competition, imposter syndrome, and academic pressure. Schools should offer stress management and resilience workshops. Professors should emphasize that one bad grade does not define a student's STEM career.
  – **Rethinking Job and Study Balance**. More on-campus employment opportunities related to STEM should be created, as being a TA/tutor has helped some students academically. Schools could introduce more paid research opportunities so students don't have to choose between income and academics.
5. Promoting effective academic skills.
  – **Enhance Mentorship and Peer Networks**. Programs that connect students with professionals in STEM could strengthen retention and engagement. Organize Peer-led study groups and mentorship initiatives should be expanded.
  – **Faculty-Driven Support and Advising**. Professors and advisors should continue offering structured academic guidance, especially in challenging STEM courses.
  – **Improve Access to Research and Internship Opportunities Without Overwhelming Students**. Schools should provide structured research opportunities so students can gain experience without feeling like they must “do it all.” Some students find research opportunities through advisors, while others struggle to access them. Schools should ensure all students know how to find and apply for research and internship programs.

## 6 Limitations

Evaluating the evidence without biases is critical to underpin practice (Smith and Noble, 2025). This study has the potential of sampling bias or potential exclusion of less-connected students, the recruitment relied on peer referrals from the RAs who are already motivated STEM students. There is the potential that less-connected students and some non-Biology/Chemistry majors may be under-represented, which could bias the sample toward the RAs' networks. Students in other STEM of life sciences disciplines may not be represented in the results of this qualitative study. Also, this study did not have the opportunity to conduct inter-rater reliability of the instrument and therefore the results may subject to threat of internal validity.

## 7 Conclusion

MSIs are tasked with creating an equitable learning environment that will be conducive to successful, academically prepared students who are equipped with the necessary skills to enter STEM career pathways. MSIs often create nurturing academic settings that cater to the unique cultural and educational needs of their students. Fewer research has studied the relationship between the background of the scientific workforce and the type of science, population, and instruction focus; and even less studied is the role institutional structures that support this science.

This research called for an intersectionality approach to mentoring and understanding of what students experience while studying at this Urban MSI. As an Urban MSI in New York City the diversity of students falls beyond race and ethnicity to include layers of immigration, inter-generational students, religion, urban versus rural commuting students, low- and high-income residents in NYC attending public institutions, high income multilingual students vs. English as a second language students coming out of NYC school districts, and more, and specially in gender differences. All of these areas merit more analyses to understand their narratives and counter narratives as they interface with the STEM studies and careers. Each of these have a relation to their parental role models, how they experience instructor mentorship, study-work balance, and the other narratives identified in this research.

## Data Availability

The raw data supporting the conclusions of this article will be made available by the authors, without undue reservation.
